# Supplementary material for: Facile Splint-Free Circularization of ssDNA with T4 DNA Ligase by Redesigning the Linear Substrate to Form an Intramolecular Dynamic Nick
Source: Biomolecules. 2024 Aug 18;14(8):1027. doi: 10.3390/biom14081027 (PMC11352879; doi:10.3390/biom14081027)
Supplement: Supplementary file 1 [file biomolecules-14-01027-s001.zip › Biomolecules-circular-ssDNA-SI.pdf]

Supporting information for

Article

Facile Splint-Free Circularization of ssDNA with T4 DNA Ligase by Redesigning the Linear Substrate to Form an Intramolecular Dynamic Nick

Wenhua Sun [1], Kunling Hu [1], Mengqin Liu [1], Jian Luo [1,†], Ran An [1,2,*]

and Xingguo Liang [1,2,*]

1 State Key Laboratory of Marine Food Processing & Safety Control, College of Food Science and Engineering, Ocean University of China, No. 1299 Sansha Road, Qingdao 266404, China

2 Laboratory for Marine Drugs and Bioproducts, Qingdao Marine Science and Technology Center, No. 1 Wenhai Road, Qingdao 266237, China

***** Correspondence: ar@ouc.edu.cn (R.A.); liangxg@ouc.edu.cn (X.L.)

† Current address: Department of Chemistry & Biotechnology, School of Engineering, The University of Tokyo, 7 Chome-3-1 Hongo, Bunkyo-ku, Tokyo 113-8654, Japan.

Supporting Table

Table S1

Name	Sequences (5′→3′)	Length (nt)
L0	ACAACGAAAGTAGCAGAAATACAGGTATCTAGGCTAGCT	39
L1	GCAGAAATACAGGTATCTAGGCTAGCTACAACGAAAGTA	39
L2	TCTTCACACATACTACACACTACTACAGGCATGAATCAGAATGAT	45
L3	TCGTTCACACATACTACACACTACTACAGGCATCGAATCAGAATGAT	47
L4	TCGATTCACACATACTACACACTACTACAGGCATTCGAATCAGAATGAT	49
L5	CGTTCACACATACTACACACTACTACAGGCATCGATCAGAATGAT	45
L6	CGGTTCACACATACTACACACTACTACAGGCATCCGATCAGAATGAT	47
L7	TCGGTTCACACATACTACACACTACTACAGGCATCCGAATCAGAATGAT	49
L8	CGTTCACACATACTACACACTACTACAGGCATCGATCGAAGAT	43
L9	CGTTCACACATACTACACACTACTACAGGCATCGGTCGAAGAC	43
L10	CGTTCACACATACTACACACTACTACAGGCATCGGGCGAAGCC	43
L11	CGTTTCGATCGAAGAT	16
L12	CGTTATCGATCGAAGAT	17
L13	CGTTCATCGATCGAAGAT	18
L14	CGTTCCATCGATCGAAGAT	19
L15	CGTTCACATCGATCGAAGAT	20
L16	CAGTCTGATAAGCTAT	16
L17	CAGTCCTGATAAGCTAT	17
L18	CAGTCTCTGATAAGCTAT	18
L19	CAGTCGTCTGATAAGCTAT	19
L20	CAGTCAGTCTGATAAGCTAT	20
L21	TCGATTCACACATACTACACACTACTACAGGCATTCGAATCGAAGAT	47
L22	GAGCTTCAGATCAGAGCCCTCT	22
L23	CACGTTCTTTAATAGTGGACCGTTGGAGTC	30
L24	TCTGATAAGCTATCAACATCAGTCTGATAAGCTATCAACATCAG	44
L25	CCCACAGCAGATGTGACTGTGAATCGTGACTCCCAATTGGGTACGCAGTA	50
L26	GCTTTCAAAAAAAATAGTCGTGTCGTGAGCAGTGAAAAAATTGAAAGCTAACGA AAACGTTA	62
L27	TCGAACACTTTCACACATACTACACACTACTACAGGCATAGTGTTCGATCGAAGA	55
L28	TCGAACACTTTCACACATACTACACACTACTACAGGCATAGTGTTCGAGCGAAGC	55
splint	CGTTGTAGCTAG	12

Table S1. The sequences of typical l-DNAs and the splint used in this study. Except for splint, all sequences are phosphorylated before ligation.

Supporting Figure

Figure S1

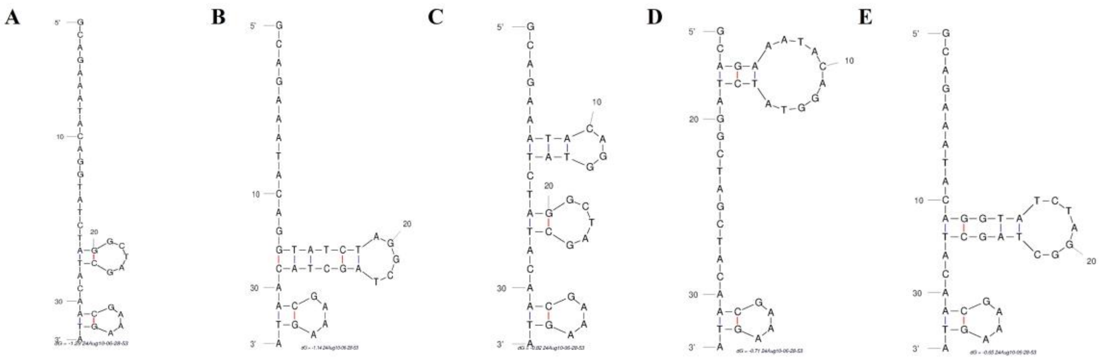

Fig. S1. Different structures of L1 simulated by Mfold. The calculation is carried out under the conditions of [Mg^{2+}] = 1 mM and 25°C.

Figure S2

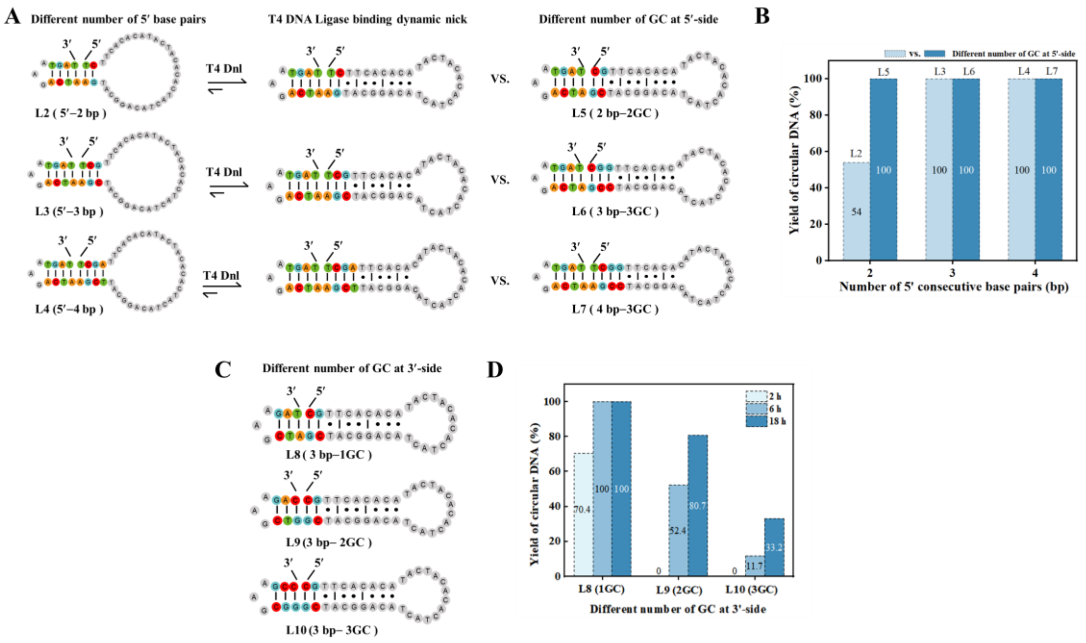

Fig. S2. Circularize ssDNA with various stem sequences**.** (A) Sequences with different consecutive base pairs at 5′-side. (B) Circularization yield for various sequences in Figure A. Conditions for the reaction: 3 μM ssDNA, 0.25 U/μL T4 Dnl in 0.1× T4 ligase buffer at 25°C for 12 h, analyzed by 12% PAGE. (C) Sequences with different consecutive base pairs at 3′-side. (D) Circularization yields for various sequences in Figure C. The conditions are the same as above.

Figure S3

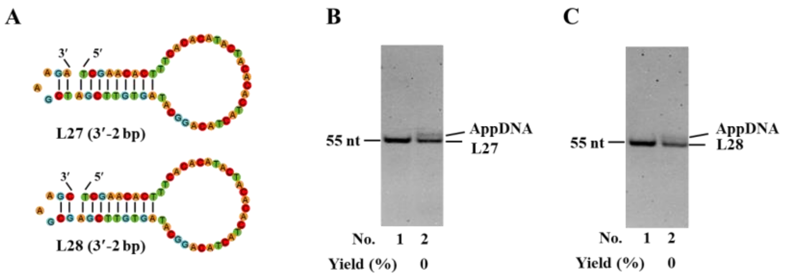

Fig. S3. The length limit for efficient circularization at the 3′-end. (A) Sequences with 2 consecutive base pairs at 3′-side. (B, C) Electrophoresis analysis for circularization of the sequences in Figure A. Lane 1, linear ssDNA (no T4 Dnl); Lane 2, L1 treated with T4 Dnl. Conditions for the reaction: 3 μM ssDNA, 0.25 U/μL T4 Dnl in 0.1× T4 ligase buffer at 25°C for 6 h, analyzed by 12% PAGE.

Figure S4

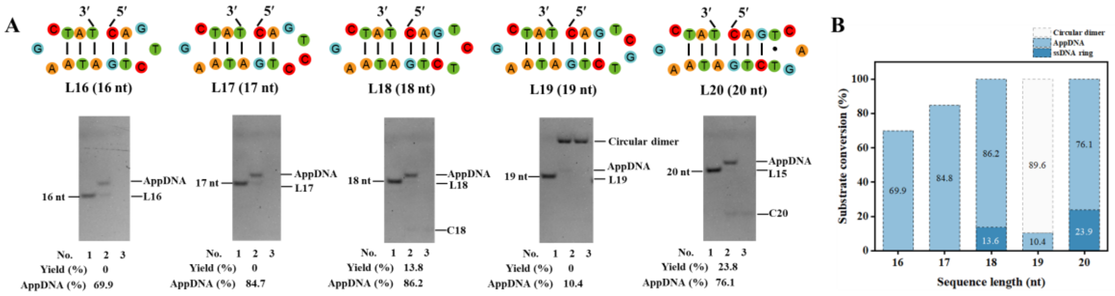

Fig. S4. The length limit for efficient circularization. (A) Sequences with different lengths at 5′-side and electrophoresis results. Lane 1, linear substrate. Lane 2, ligation by T4 Dnl. Lane 3, the products in Lane 2 were treated with Exonuclease I and Exonuclease III. (B) Circularization and adenylation yield for various sequences in Figure A. Conditions for the reaction: 0.6 μM ssDNA, 0.25 U/μL T4 DNA ligase in 0.1× T4 ligase buffer at 25°C for 18 h, analyzed by 12% PAGE.

Figure S5

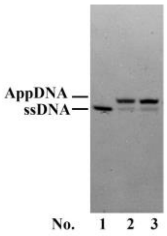

Fig. S5. Circularization of n1 without ATP after adenylation. Lane 1, linear ssDNA (n1) without T4 DNA ligase. Lane 2, the linear ssDNA (n1) treated with T4 DNA ligase. Lane 3, lane 2 removed the ATP and retreated with T4 DNA ligase without ATP at 25°C for 12 h. Analyzed by 12% PAGE.

Figure S6

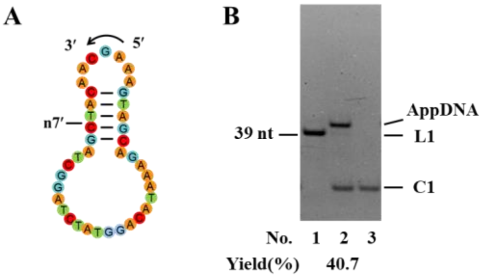

Fig. S6. Circularization result of n7′. (A) The diagram of different sequence direction of n7 named n7′ (reverse 5′→3′ to 3′→5′ without changing the bases). (B) Electrophoresis analysis for circularization of n7′. Lane 1, linear ssDNA (n7′) with no T4 Dnl. Lane 2, L1 treated with T4 Dnl. Lane 3, the products in Lane 2 were further treated with Exonuclease I and Exonuclease III. Circularization conditions: 0.6 μM ssDNA, 0.25 U/μL T4 DNA ligase in 0.1× T4 ligase buffer at 25°C for 18 h. Analyzed by 12% PAGE.

Figure S7

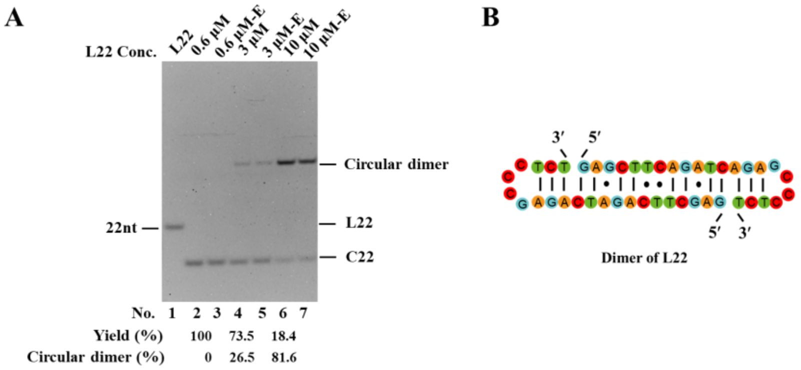

Fig. S7. Effect of concentration on L22. (A) Electrophoresis analysis for circularization of L22. Lane1, L22 without the T4 ligase treatment. Lane 2, lane 4 and lane 6, different concentrations of L22 treated with T4 DNA ligase. Lane 3, lane 5 and lane 7, the products in lane 2, lane 4 and lane 6 were further treated with Exonuclease I and Exonuclease III. Conditions: L22 with different concentrations, 0.5 U/μL T4 DNA ligase in 0.1× T4 ligase buffer at 37°C for 12 h. Analyzed by 12% PAGE. (B) Possible secondary structure of the dimer of L22.

Figure S8

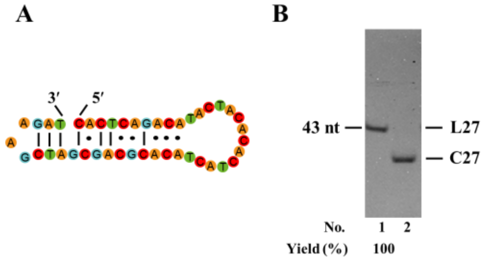

Fig. S8. Effect of mismatch at the second adjacent location of nick. Lane1, L27 without the T4 ligase treatment. Lane 2, L27 treated with T4 DNA ligase. The reaction conditions: 0.6 μM linear ssDNA, and 0.5 U/μL T4 DNA ligase in 0.1× T4 ligase buffer, 25°C, 6 h. Analyze with 12% PAGE.

Figure S9

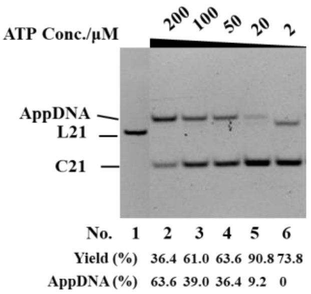

Fig. S9. Effect of the concentration of ATP. Lane 1, L21 without T4 ligase treatment. Lane 2–6, L21 treated with T4 DNA ligase with different concentrations of ATP. The reaction conditions: 1 μM L21, 0.5 U/μL T4 DNA ligase in 1× T4 ligase buffer (without ATP) and different concentrations of ATP at 25°C for 18 h. Analyzed by 12% PAGE.
