# Supplementary figures and images for: Facile Splint-Free Circularization of ssDNA with T4 DNA Ligase by Redesigning the Linear Substrate to Form an Intramolecular Dynamic Nick
Source: Biomolecules. 2024 Aug 18;14(8):1027. doi: 10.3390/biom14081027 (PMC11352879; doi:10.3390/biom14081027)

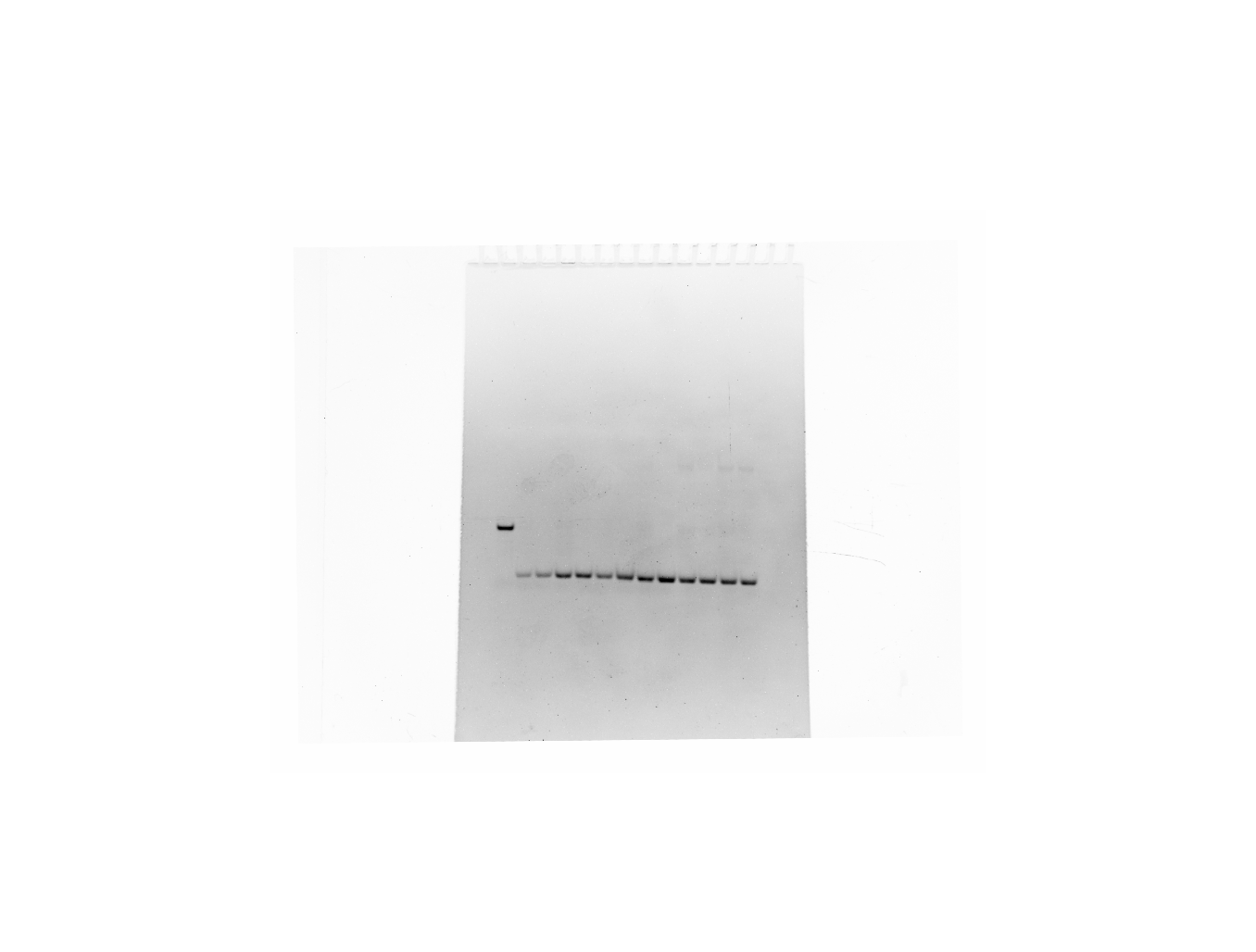

Supplement: Supplementary file 1 [file biomolecules-14-01027-s001.zip › origin image/Fig.1D.tif]

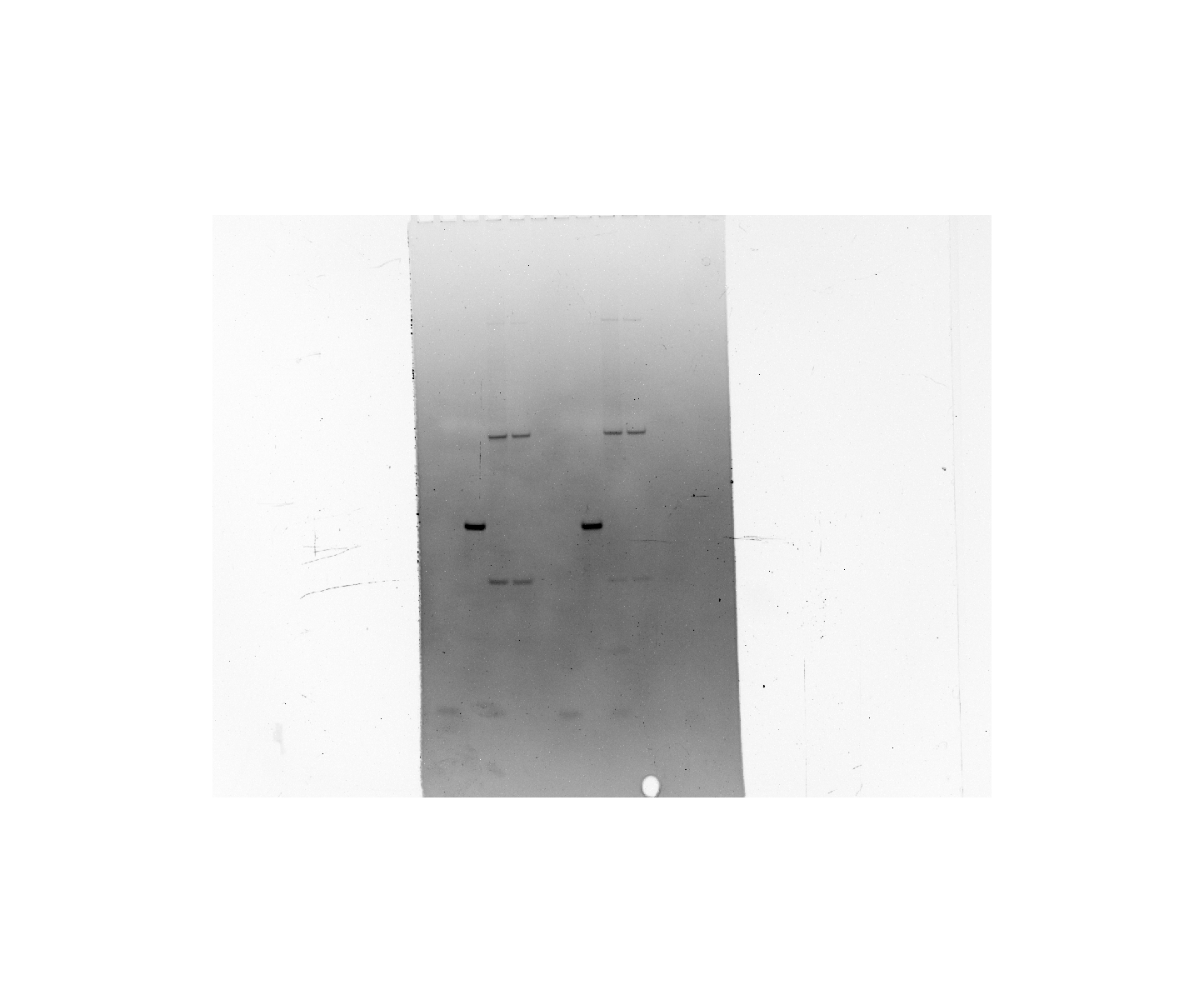

Supplement: Supplementary file 1 [file biomolecules-14-01027-s001.zip › origin image/Fig.1E.tif]

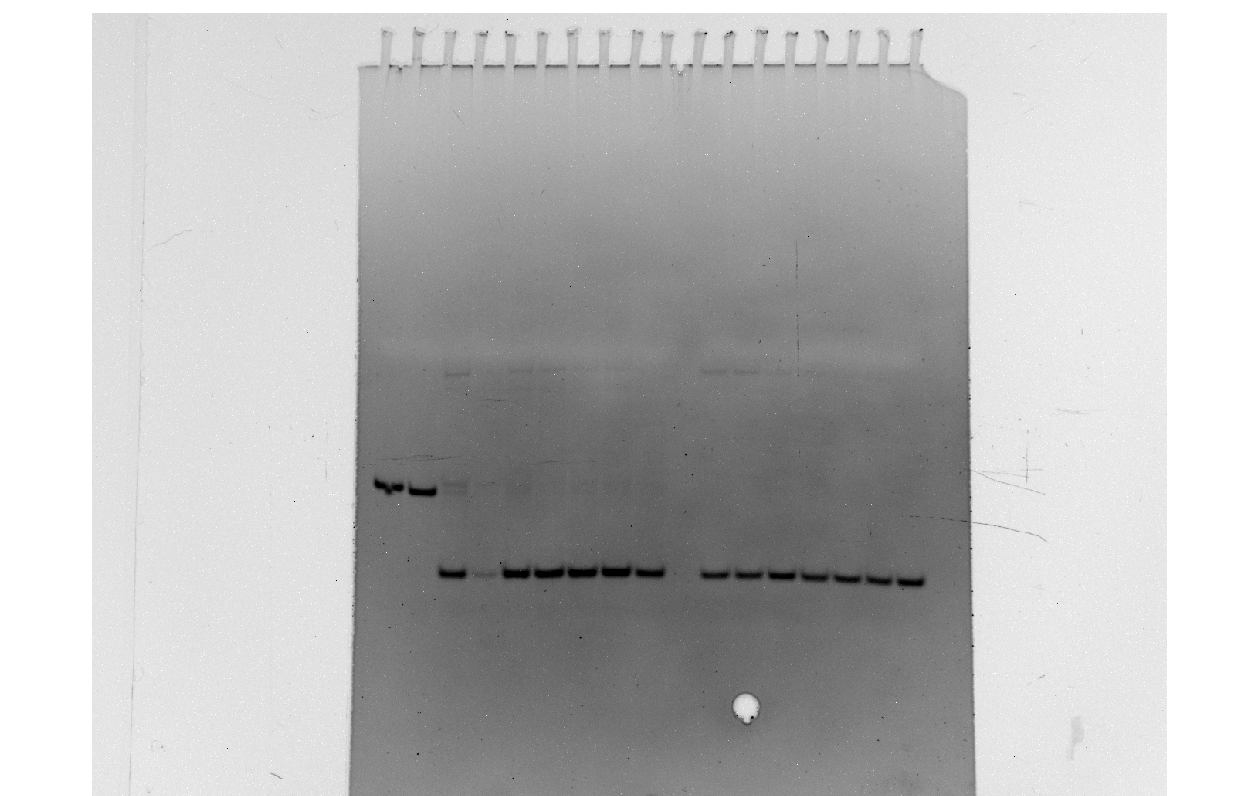

Supplement: Supplementary file 1 [file biomolecules-14-01027-s001.zip › origin image/Fig.1F.tif]

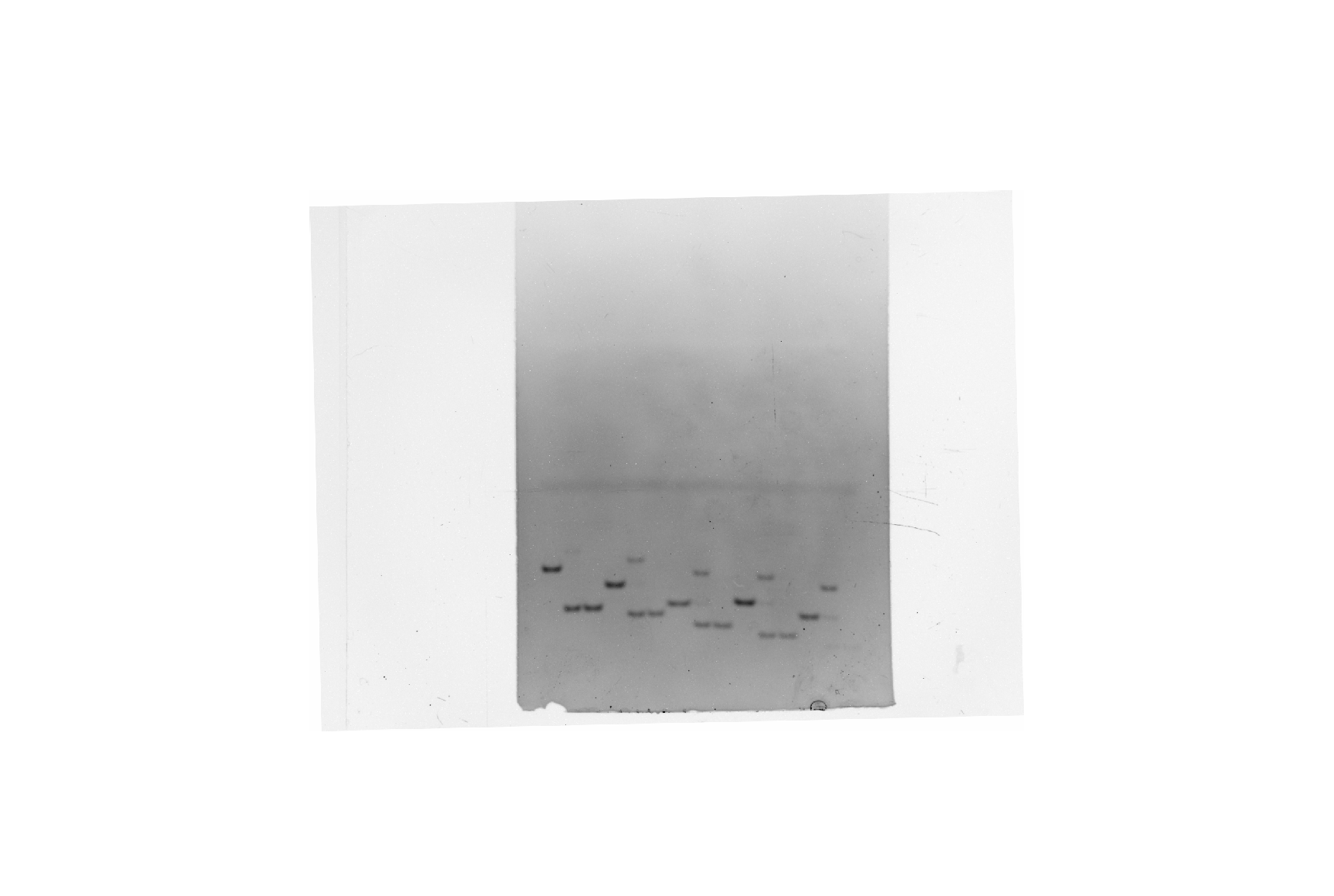

Supplement: Supplementary file 1 [file biomolecules-14-01027-s001.zip › origin image/Fig.2.tif]

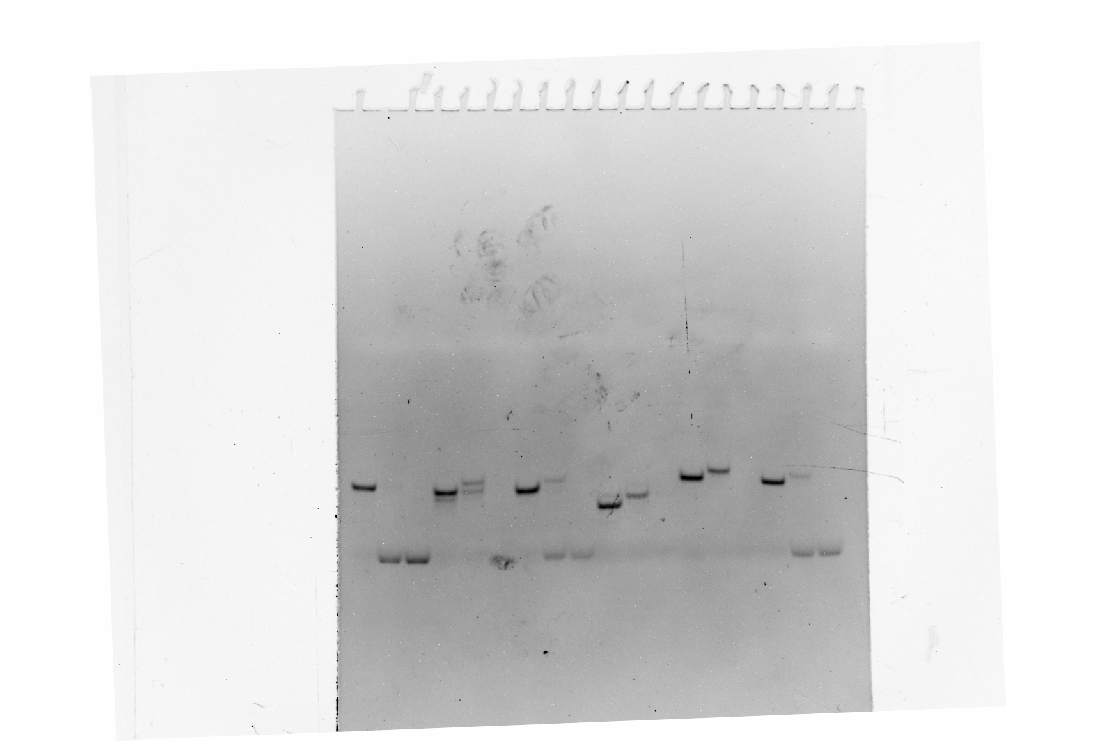

Supplement: Supplementary file 1 [file biomolecules-14-01027-s001.zip › origin image/Fig.3B(1).tif]

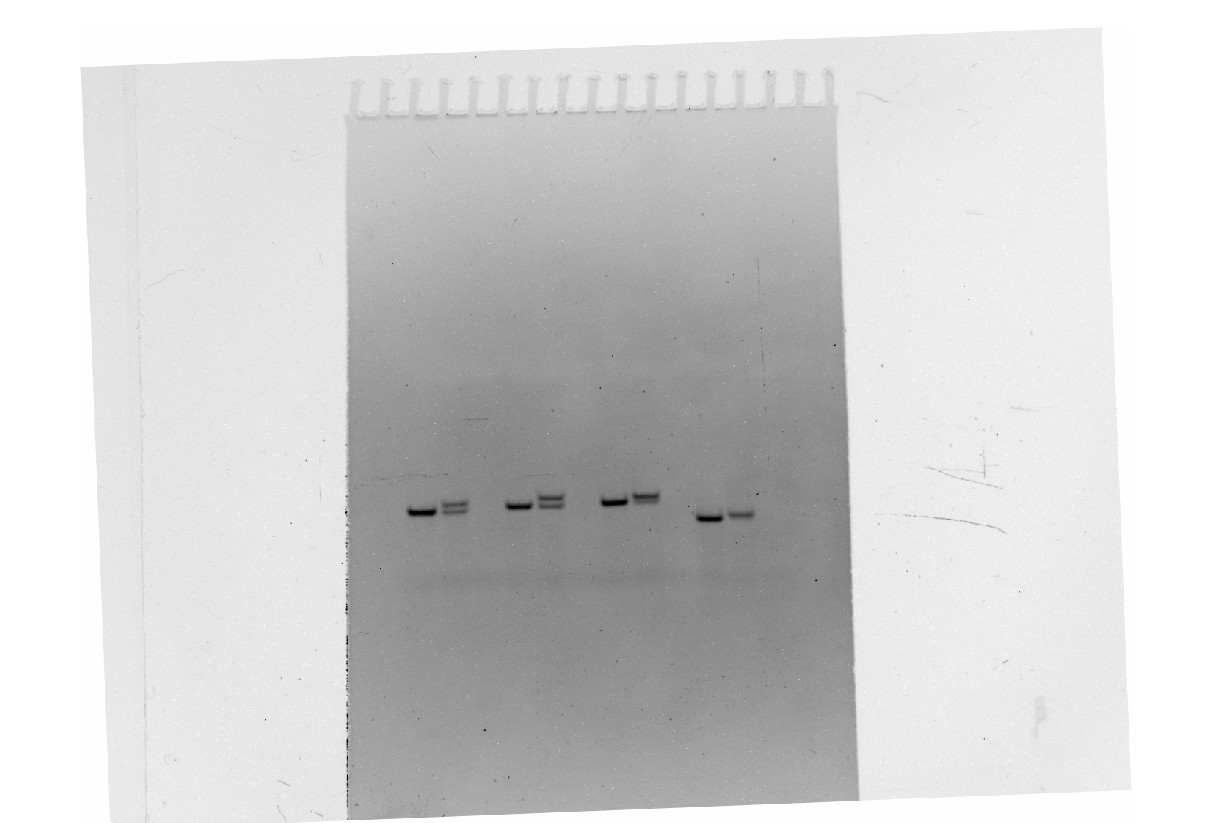

Supplement: Supplementary file 1 [file biomolecules-14-01027-s001.zip › origin image/Fig.3B(2).tif]

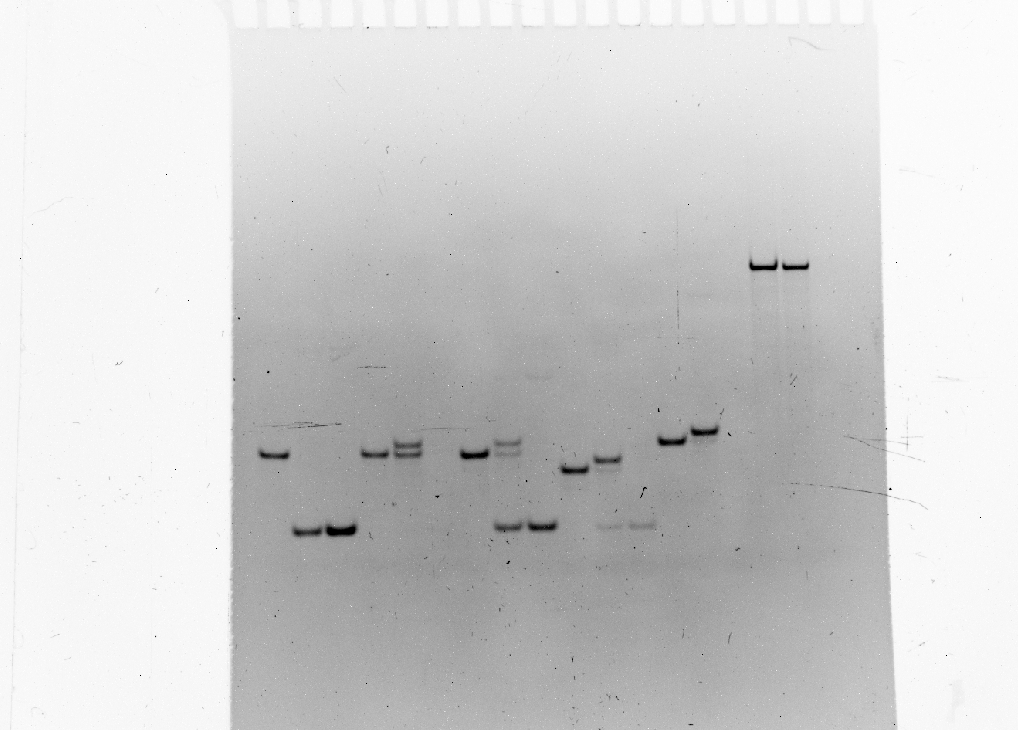

Supplement: Supplementary file 1 [file biomolecules-14-01027-s001.zip › origin image/Fig.3C(1).tif]

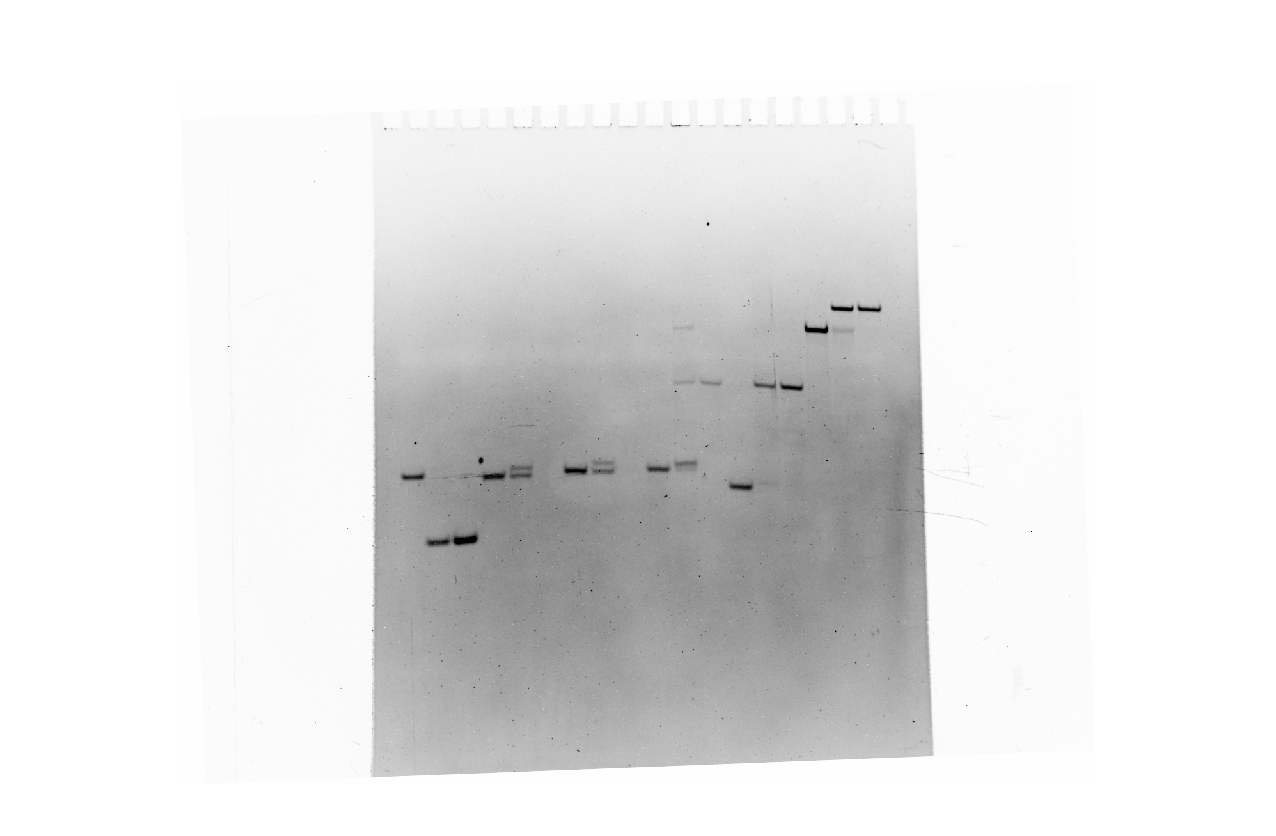

Supplement: Supplementary file 1 [file biomolecules-14-01027-s001.zip › origin image/Fig.3C(2).tif]

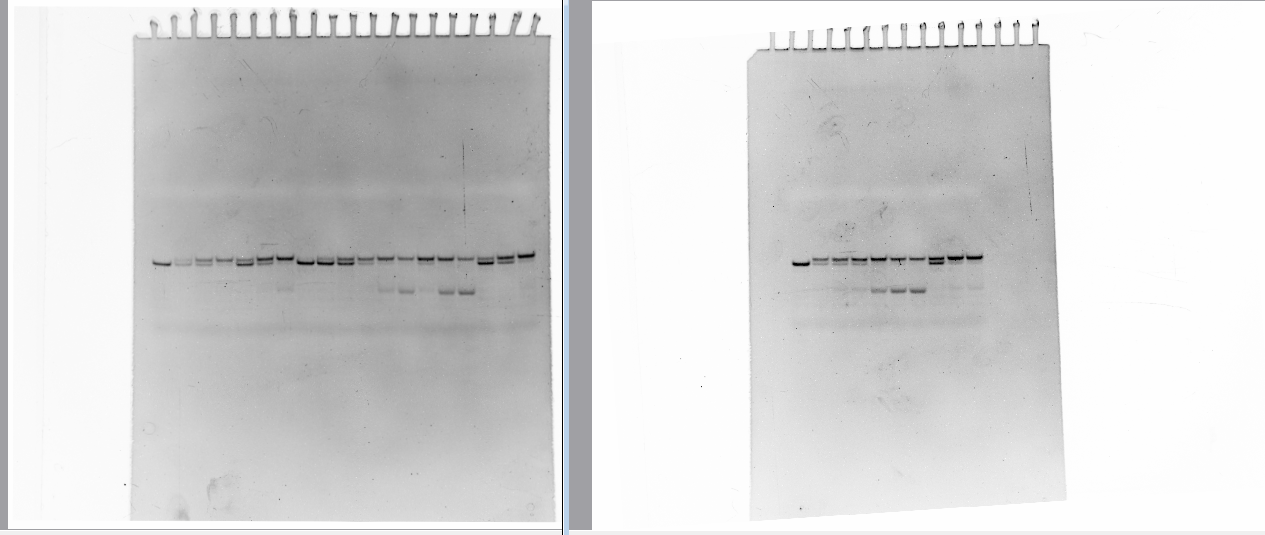

Supplement: Supplementary file 1 [file biomolecules-14-01027-s001.zip › origin image/Fig.4.tif]

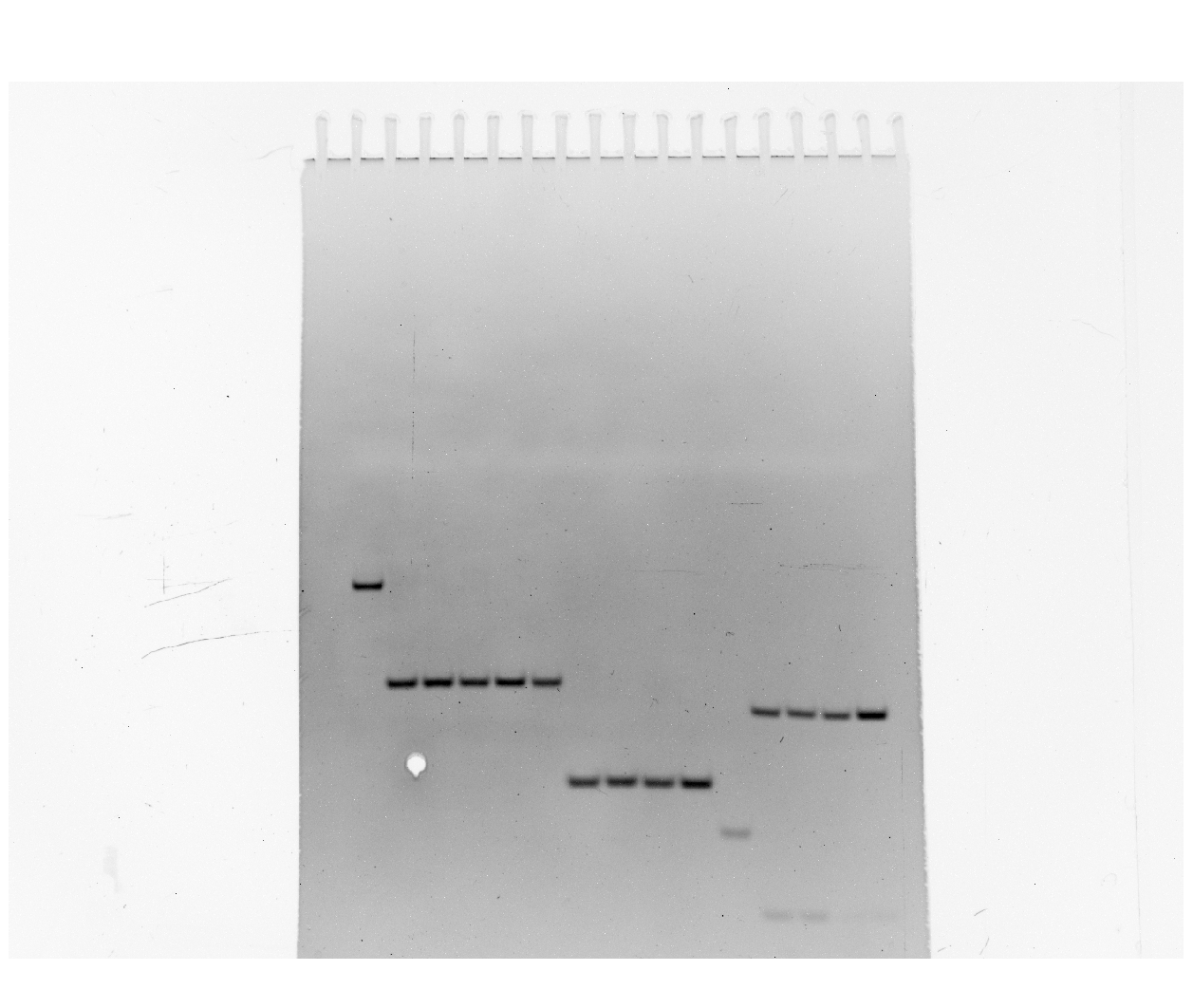

Supplement: Supplementary file 1 [file biomolecules-14-01027-s001.zip › origin image/Fig.5A,5B.tif]

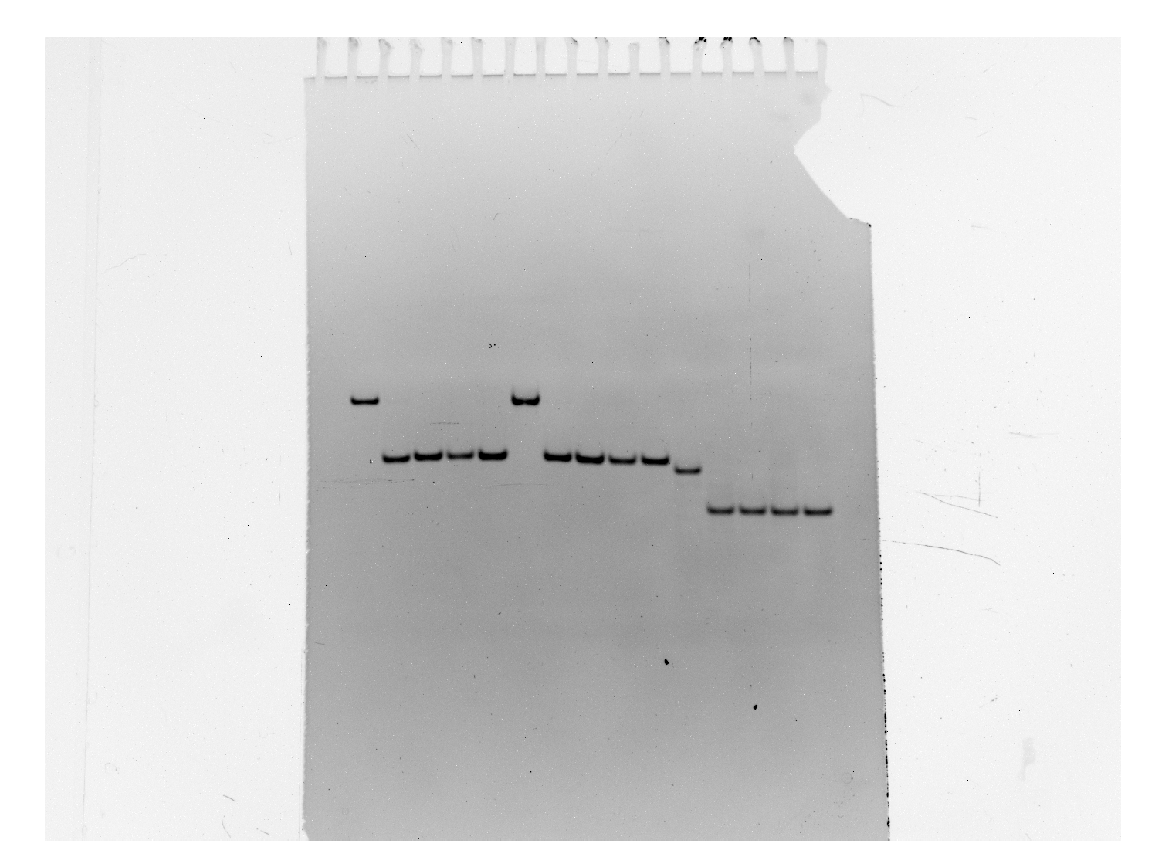

Supplement: Supplementary file 1 [file biomolecules-14-01027-s001.zip › origin image/Fig.5C,5D,5E.tif]

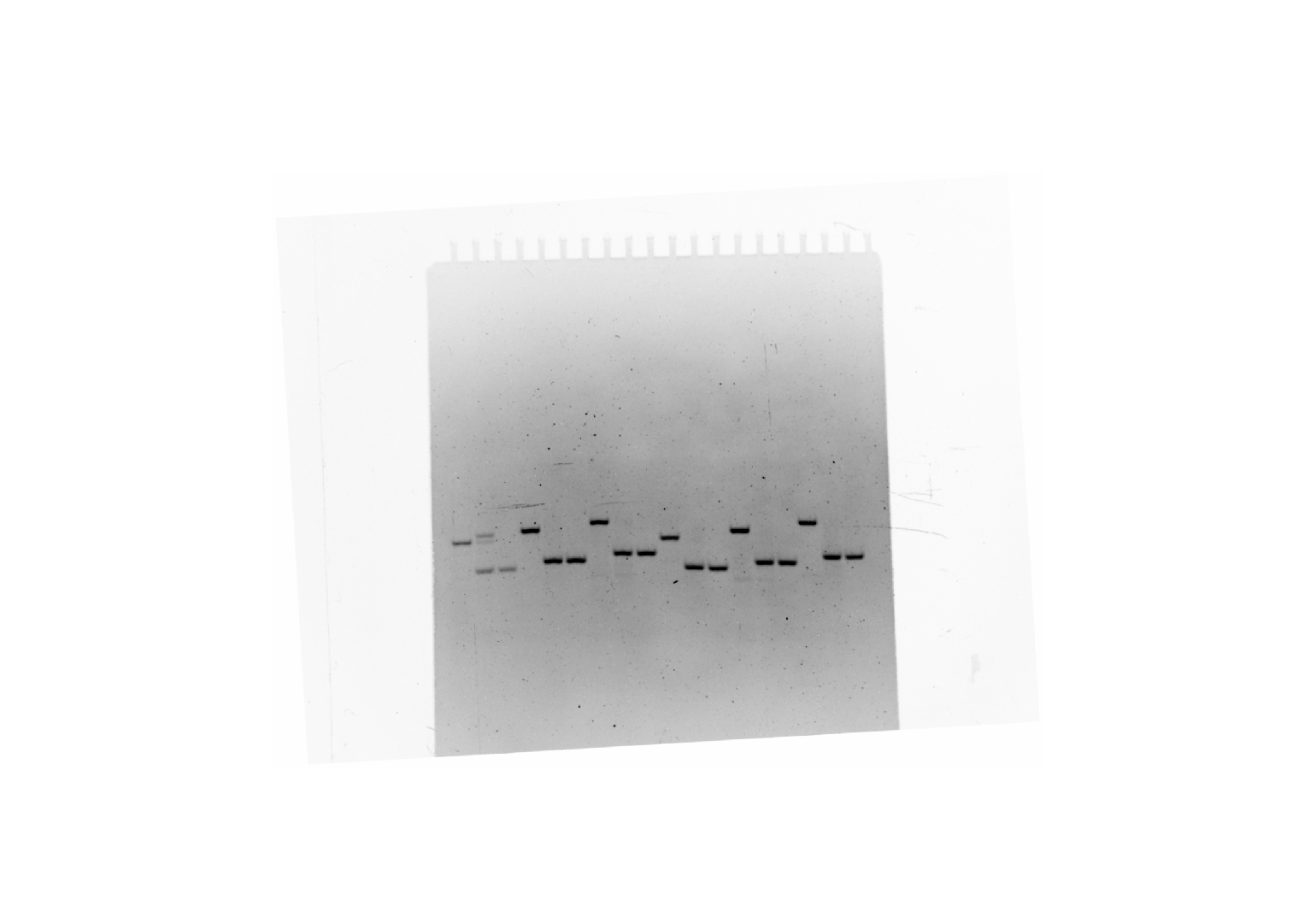

Supplement: Supplementary file 1 [file biomolecules-14-01027-s001.zip › origin image/Fig.S2B.tif]

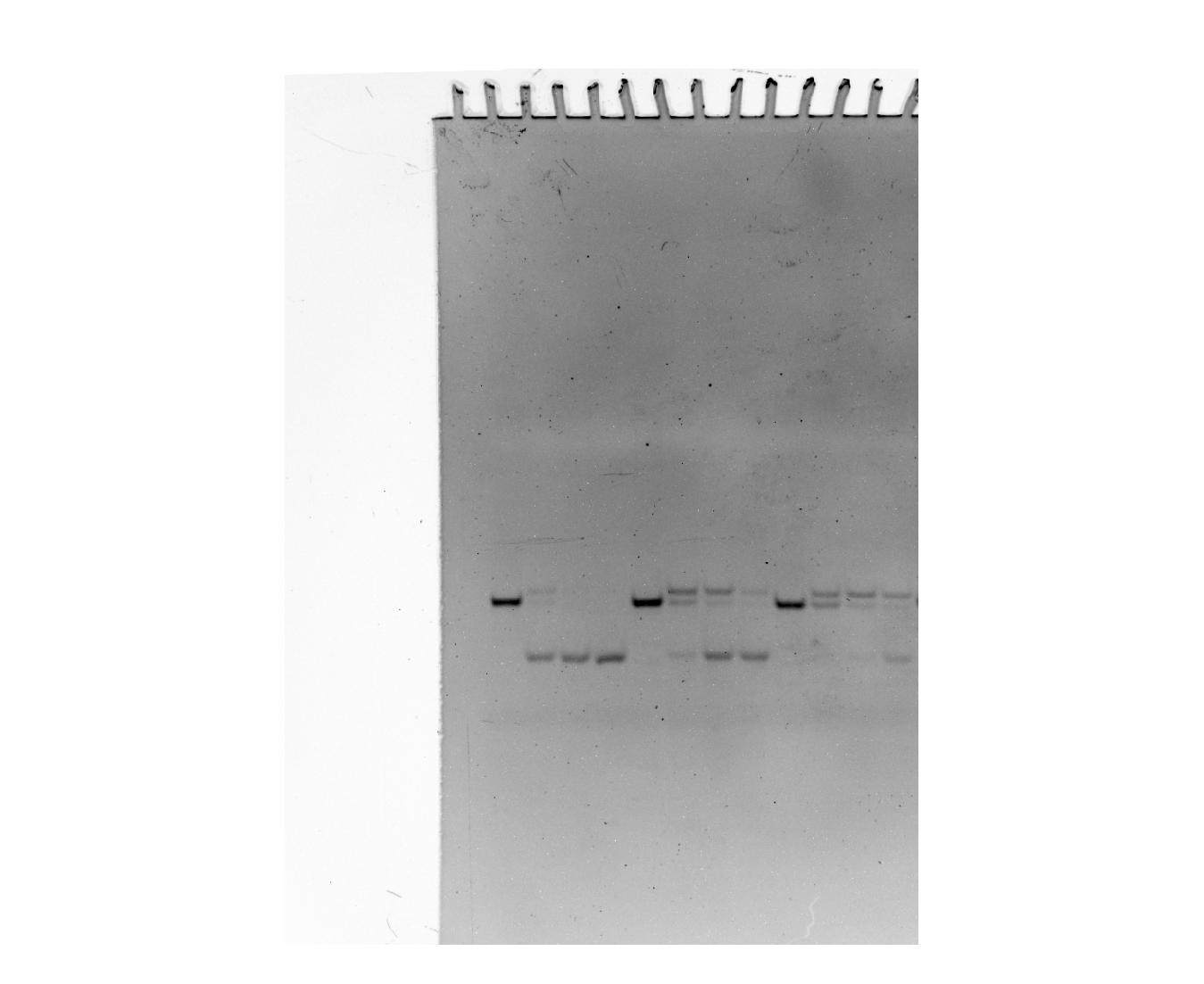

Supplement: Supplementary file 1 [file biomolecules-14-01027-s001.zip › origin image/Fig.S2D.tif]

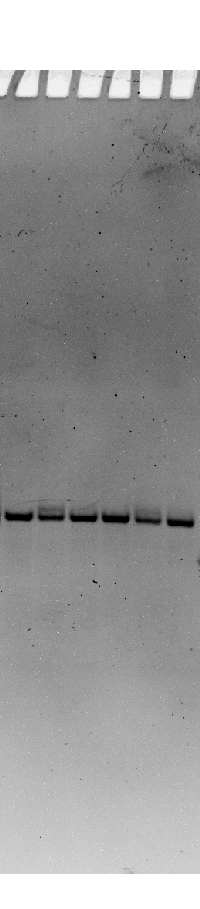

Supplement: Supplementary file 1 [file biomolecules-14-01027-s001.zip › origin image/Fig.S3.png]

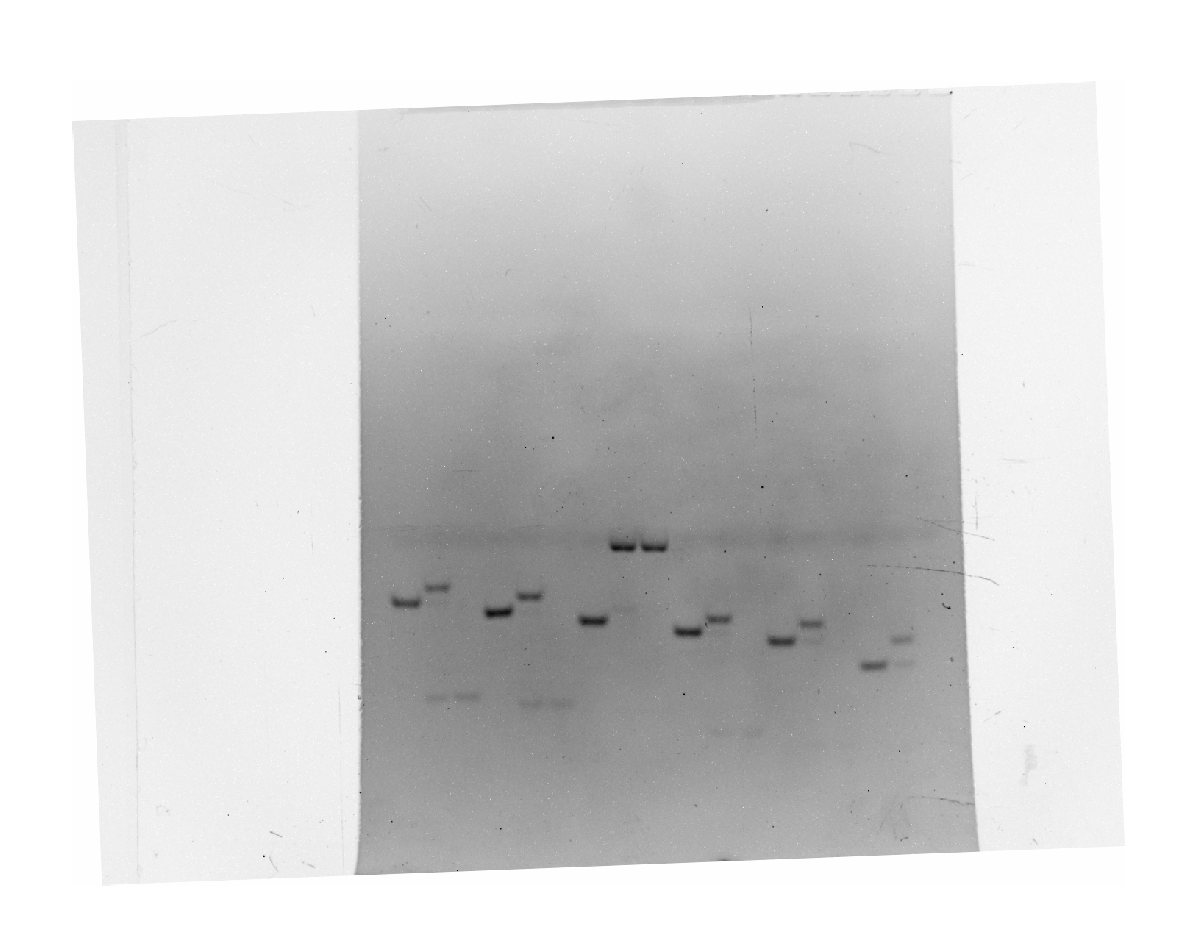

Supplement: Supplementary file 1 [file biomolecules-14-01027-s001.zip › origin image/Fig.S4.tif]

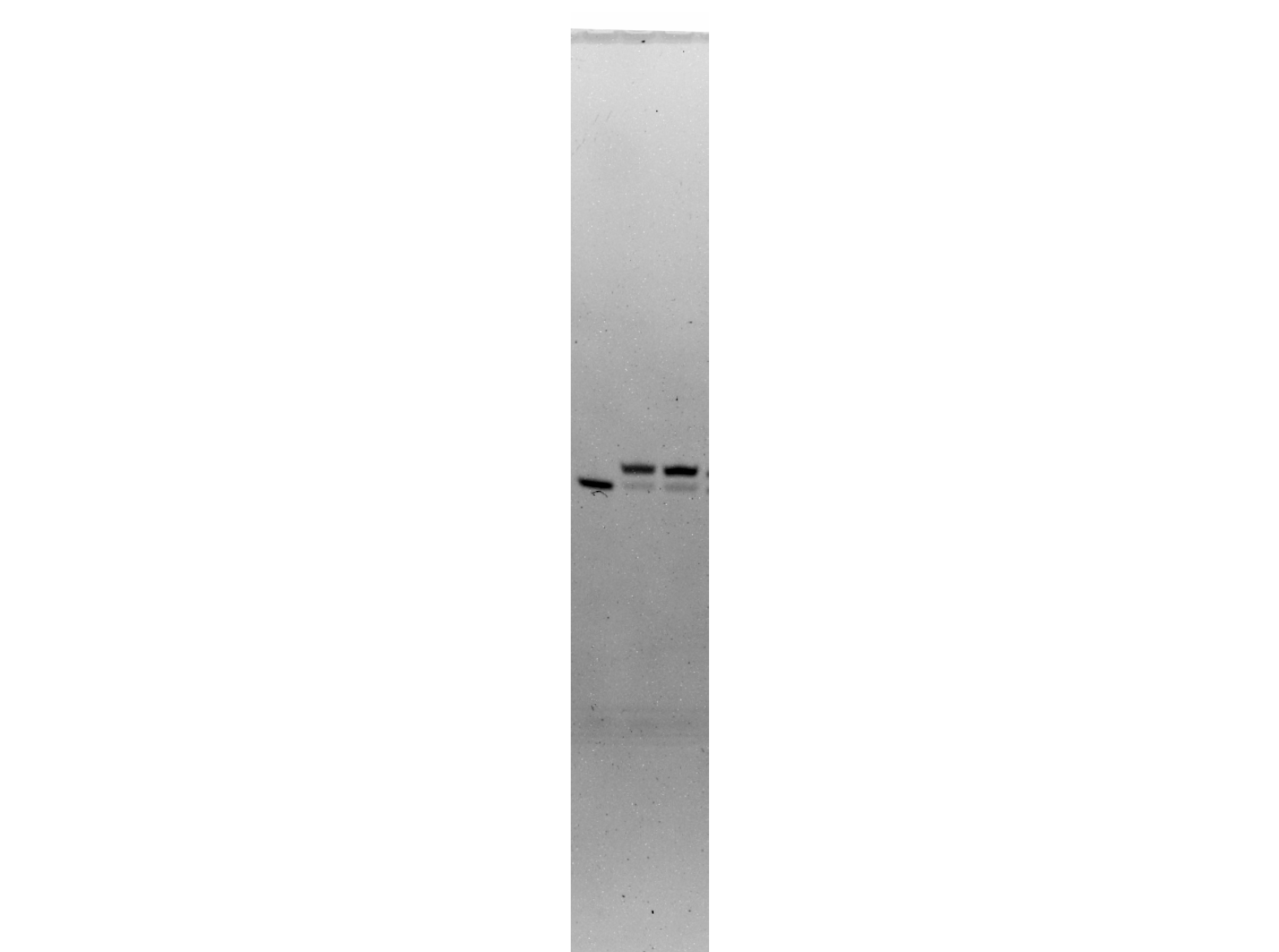

Supplement: Supplementary file 1 [file biomolecules-14-01027-s001.zip › origin image/Fig.S5.tif]

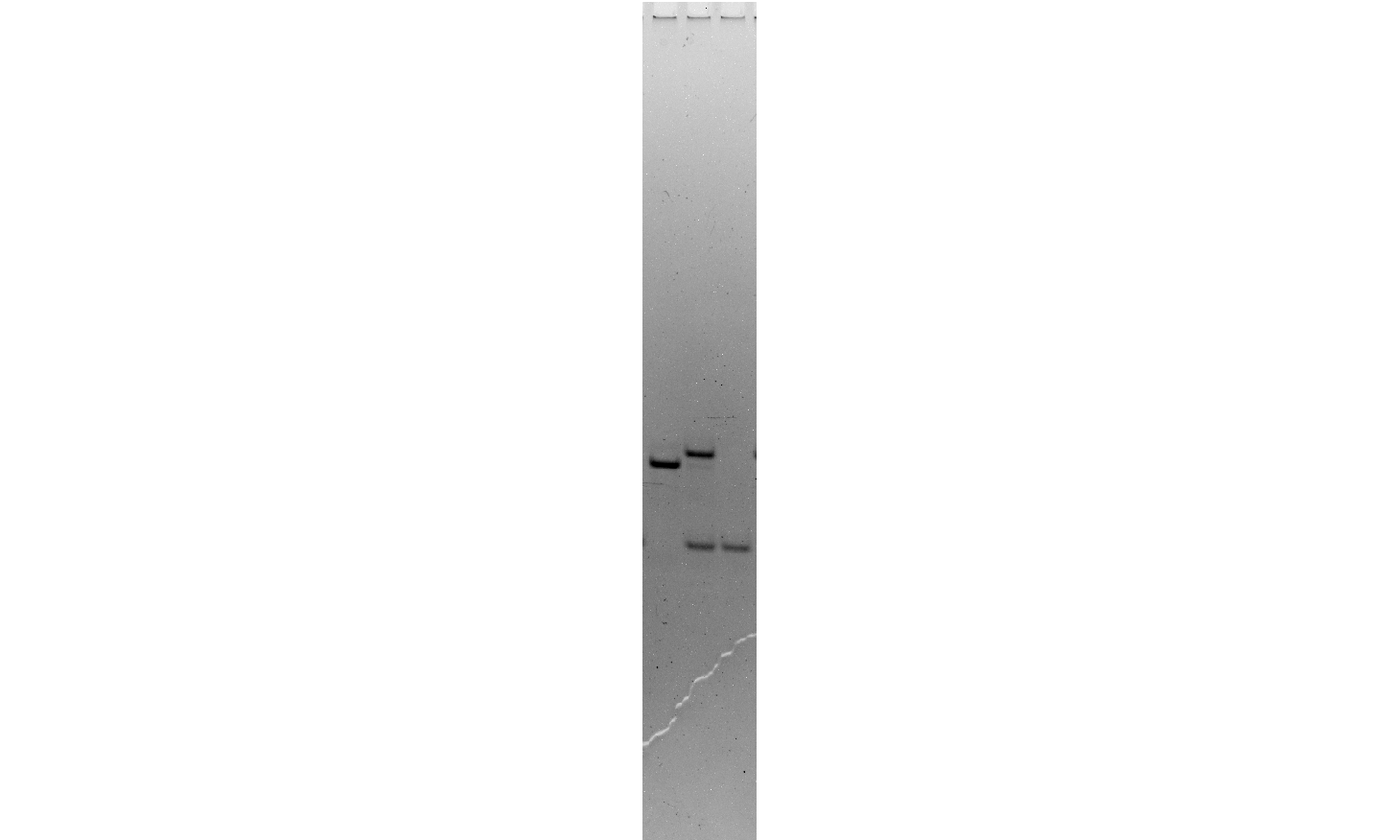

Supplement: Supplementary file 1 [file biomolecules-14-01027-s001.zip › origin image/Fig.S6.tif]

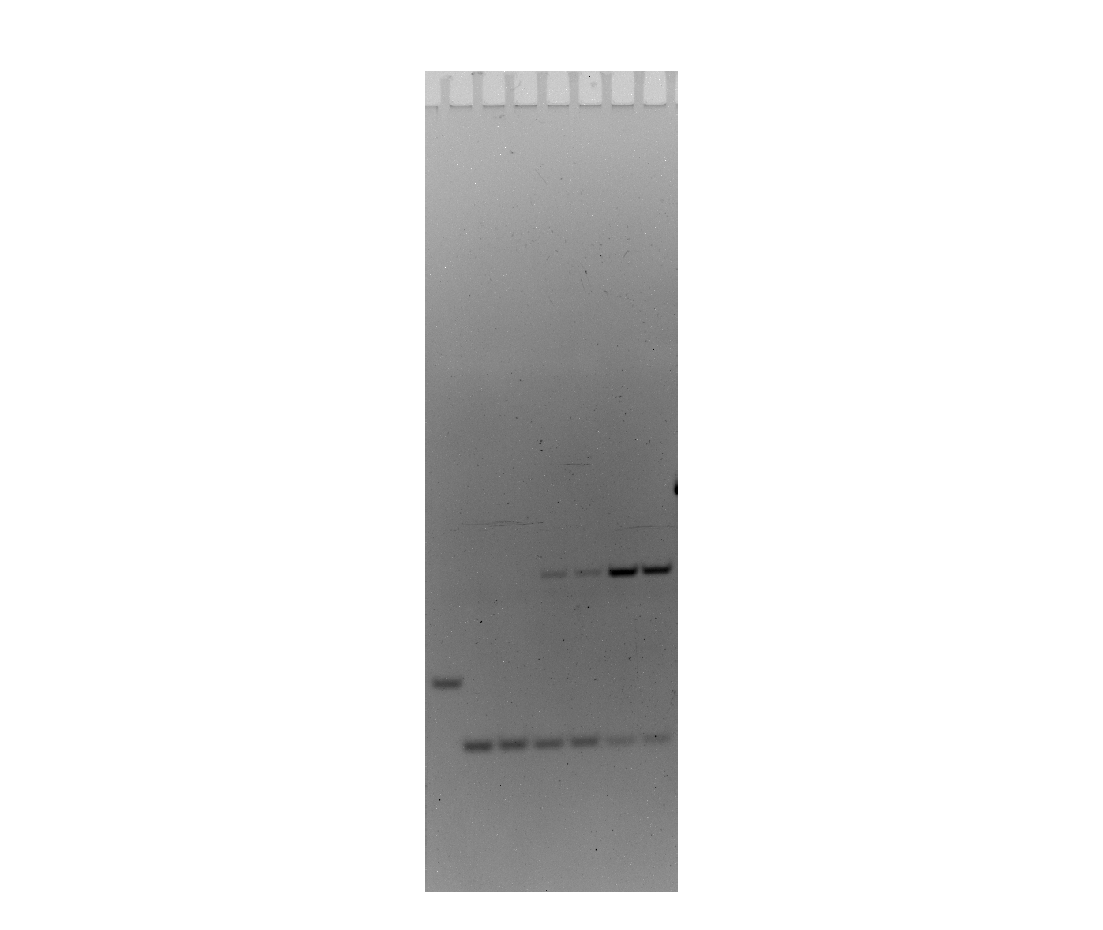

Supplement: Supplementary file 1 [file biomolecules-14-01027-s001.zip › origin image/Fig.S7.tif]

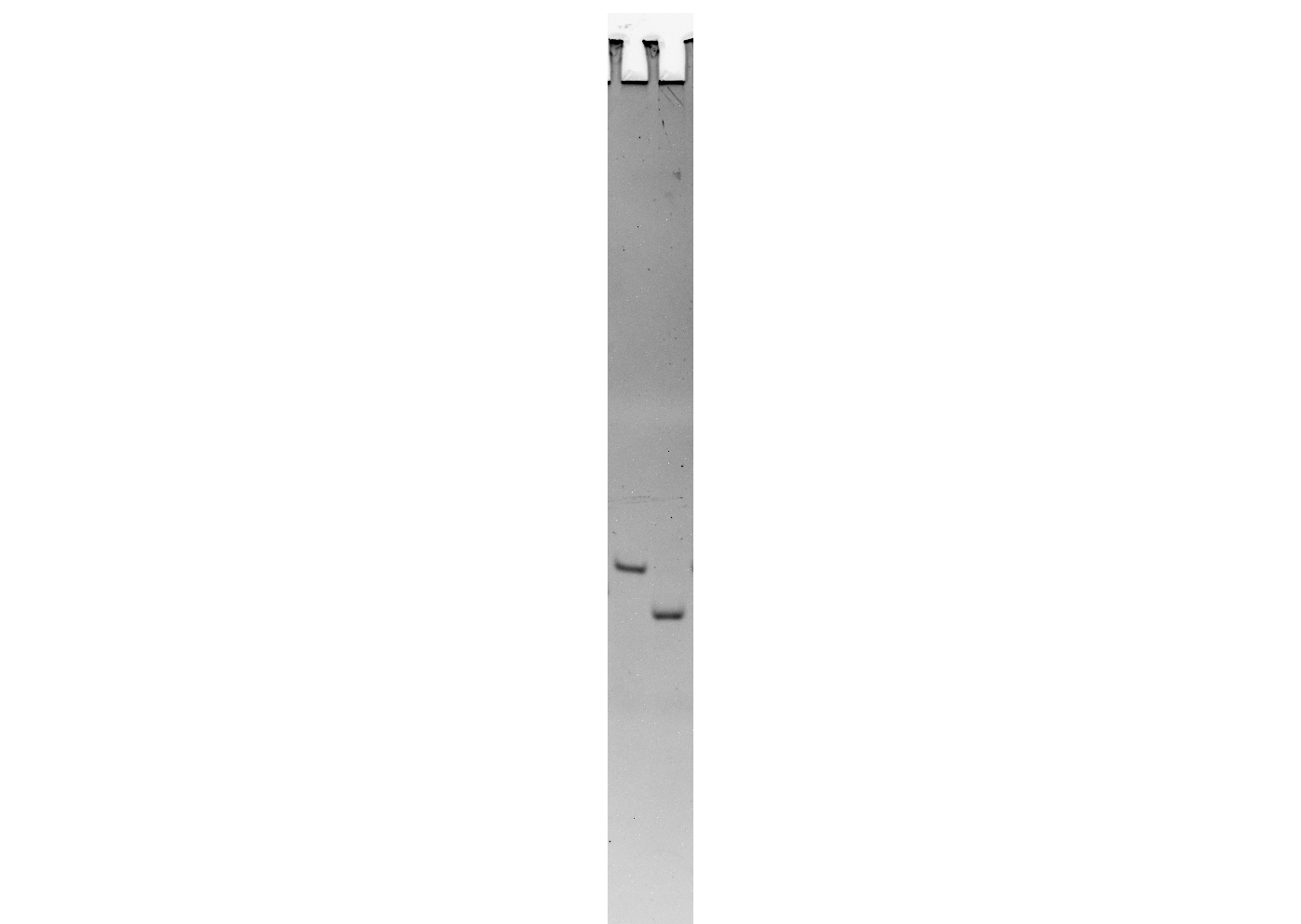

Supplement: Supplementary file 1 [file biomolecules-14-01027-s001.zip › origin image/Fig.S8.tif]

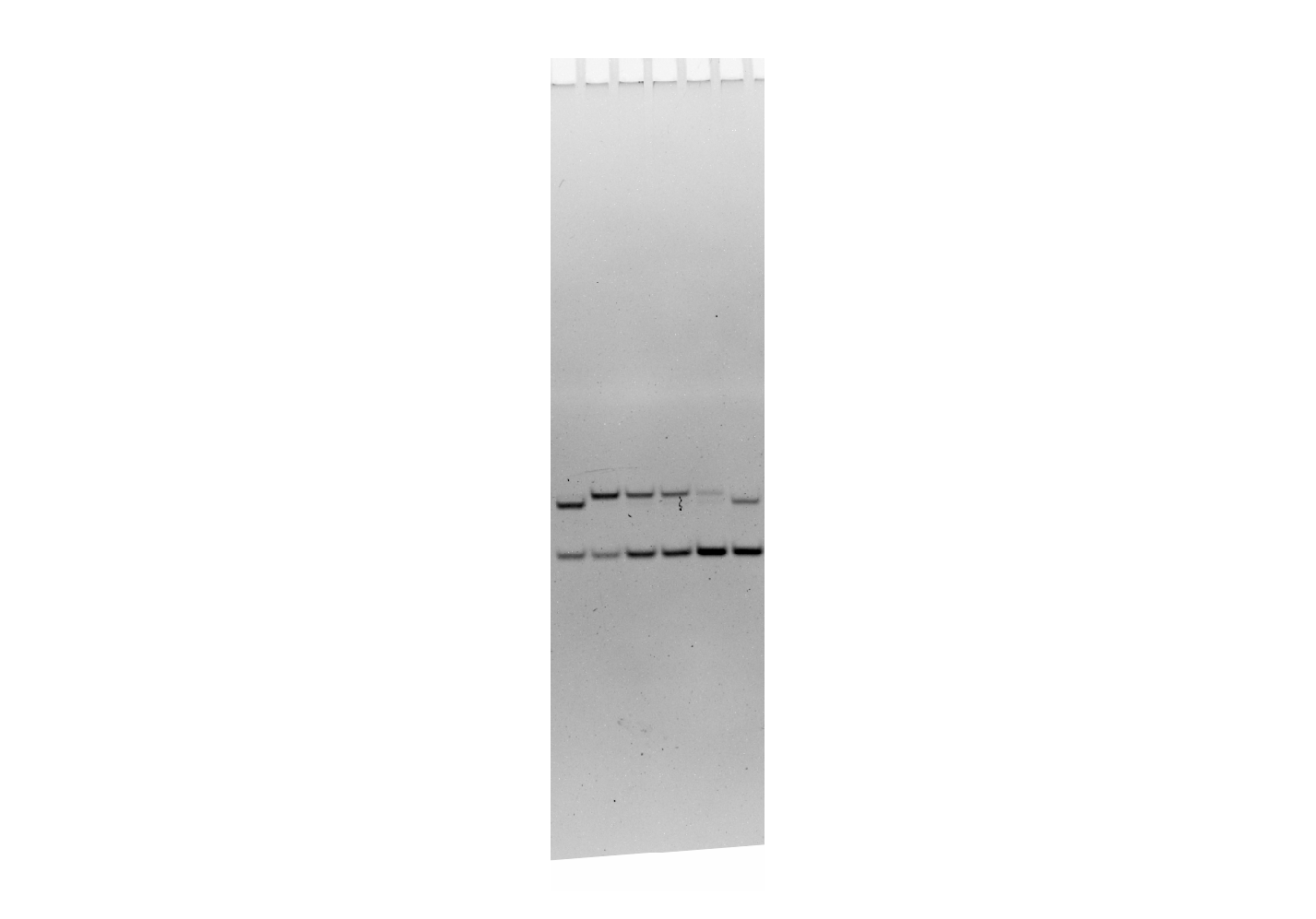

Supplement: Supplementary file 1 [file biomolecules-14-01027-s001.zip › origin image/Fig.S9.tif]
